# Differences in Adolescent’s Cardiometabolic Health: A Comparison Regarding Guided Team and Endurance Sports

**DOI:** 10.3390/ijerph192417070

**Published:** 2022-12-19

**Authors:** Ștefan Adrian Martin, Roxana Maria Martin-Hadmaș

**Affiliations:** 1 Department of Physiology, Center for Advanced Medical and Pharmaceutical Research “George Emil Palade” University of Medicine, Pharmacy, Science and Technology of Târgu Mureș, Gheorghe Marinescu 38, 540139 Mureș, Romania; 2 Department of Community Nutrition and Food Safety, “George Emil Palade” University of Medicine, Pharmacy, Science and Technology from Târgu Mureș, Gheorghe Marinescu 38, 540139 Mureș, Romania

**Keywords:** physical activity, physical training, body composition, physical fitness, energy expenditure

## Abstract

Physical exercise can induce changes in gas exchange and ventilation kinetics. Of these, some will lead to various adaptations which can influence performance and health. We conducted a single-center observational study on 40 participants. Of them, 20 participants came from volleyball (Sample 1) and the other 20 participants came from triathlon (Sample 2). All participants underwent anthropometric and basal metabolic rate measurements, along with a laboratory aerobic capacity test (VO_2peak_). In Sample 1, the VO_2peak_ was 2.38 L/min and 37.5 mL/min/kg, unlike in Sample 2, which had 2.31 L/min (*p* = 0.402, Mann–Whitney U = 204) and 43 mL/min/kg oxygen uptake (*p* = 0.0001). VE/VO_2_ was significantly different (*p* = 0.0002, Mann–Whitney U = 80), reaching 31.3 and 36.1 L/min in Sample 1 and Sample 2. Similar results were obtained regarding VE/VCO_2_ (*p* = 0.0074, Mann–Whitney U = 126), i.e., 31.3 and 33.9 L/min in Sample 1 and Sample 2. The contribution of physical activity was observed in both groups by taking into account the peak oxygen uptake. In comparison, the team sports sample showed an increased metabolic cost at the VO_2peak_.

## 1. Introduction

Regardless of age and gender, physical exercise can both positively and negatively affect the human physiological systems and the development of main movement skills. This is the reason why some physiological variables such as aerobic power, resting heart rate, breathing rate, and vital capacity may differ in individuals, as seen in early research regarding sports training, physical activity, or child physical development [1,2].

Early research papers studied exercise capacity in children and adolescents [3], and of the existing ones, some included methods that measured resistance, speed, and strength [4]. However, many of these papers researched the learning stage or the possibility to master basic motor activities, and only a few are related to endurance development.

There is a lack of evidence regarding exercise training and the physiological outcome of young athletes [5,6]. Papers that included adolescent participants measured aerobic power due to its importance as a peripheral factor in physical performance during various forms of exercise. Next to aerobic capacity, the bioenergetic stores and the hydration status, along with the intramuscular pH and the level of metabolites, influence physical exercise performance. However, they have rarely been considered in children and adolescents [7]. Moreover, using these methods, only some papers have deepened the means of physical education and sports while studying the development of exercise capacity in children [8,9,10,11] following a day-by-day training program [11,12,13].

Training can improve exercise capacity and therefore health [14,15]. Some research papers proved that aerobic physical exercise increases oxygen uptake and skeletal muscle function, while also enhancing the ability of the cardiac muscle to contract and the strength of the intercostal muscles [16]. However, anaerobic exercise was early on related to changes in muscle strength and resistance, while also increasing the risk of overtraining due to an excessive high-intensity overload [17,18]. Despite all of that, both aerobic and anaerobic physical exercise can increase the risk of overload and the risk of underdevelopment if not implemented properly in both children and adults [19]. On the other side, physical exercise will influence spatial coordination and orientation [20,21], next to muscular strength, speed, and aerobic resistance [22]. However, the motor abilities of young individuals are closely related to the physiological state, namely cardiac, respiratory, muscular, and nervous systems, along with height, body weight, body mass, and the level of physical exercise.

A lower-than-normal aerobic capacity is nowadays seen due to lack of exercise or improper planning [23]. The short- and medium-term consequences are already very well-known and refer to an increased risk of metabolic, cardiac, and respiratory clinical outcomes, while changes are already seen in their anthropometric development. In children and adolescents, it is well-known that, in recent years, there has been an increase in fat mass and a decrease in fat-free body mass [24]. In addition to these consequences, which can involve clinical manifestations, training can increase the rate of sports abandonment, which limits the number of young participants in all sports.

By measuring differences in exercise capacity, it becomes necessary to understand how the main motor qualities develop in adolescents who are enrolled in physical exercise and sports training, regardless of its type [25,26]. The qualities required to practice a particular high-performance sport can be different. This hypothesis is supported by sports training, which, in team sports, develops endurance, speed, and technical elements, compared to half-distance or long-distance events, which will develop strength and aerobic power. Therefore, our study’s aim was to evaluate the development of aerobic capacity and the ability to exercise according to the training level of two female adolescent study samples, including a volleyball and a triathlon study group. Based on our hypothesis, physical exercise can negatively affect the development of general motor skills by misdirecting physical exercise and by increasing the risk of overload. From a practical perspective, we could expect differences in oxygen uptake and carbon dioxide production during similar physical-exercise intensities. These differences will lead to various adaptations that will to a greater or lesser extent influence performance, health, and probably the state of health during the adulthood period.

## 2. Materials and Methods

### 2.1. Study Design

We conducted a single-center observational study between May 2021 and December 2021. All study procedures were held in the Center for Advanced Medical and Pharmaceutical Research, part of “George Emil Palade” University of Medicine, Pharmacy, Science and Technology of Targu Mures, Romania. The study participants underwent two stages of functional explorations. All procedures were conducted following the approval of the Ethics Committee no. 259/14.11.2018 of “George Emil Palade”, University of Medicine, Pharmacy, Science and Technology of Targu Mures, Romania.

### 2.2. Study Participants

To recruit participants, we launched a public announcement to local sports clubs wherein we presented the objective, inclusion criteria, means, and methods of research, next to the advantages, and disadvantages of the program. Before inclusion, all procedures were explained to the participants and their caregivers. Informed-consent forms were made available to them, and the legal guardian signed if the participants were to be enrolled. The withdrawal from the study was possible at any time, with prior notice.

**Figure 1:**
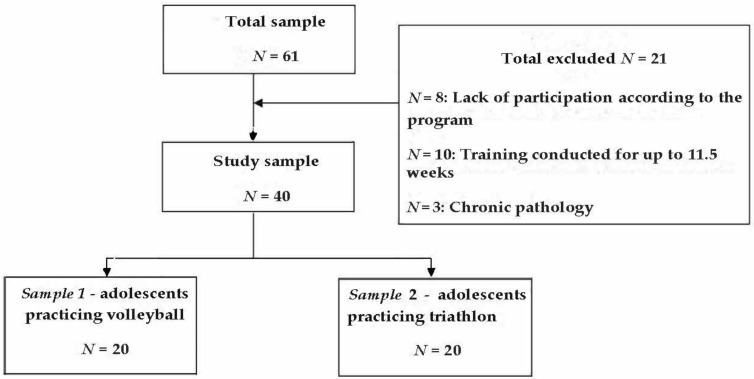
Flowchart of the study sample.

The study sample was made up of 40 female adolescent participants. To be recruited in the study, the participants had to meet the following criteria: age between 14 and 16 years, female participant, and an active member of the sports club for over 12 weeks, with weekly participation in organized physical exercise training and with at least 3 training sessions per week and a minimum volume of 180 min/week, without chronic pathologies. If any of the above criteria were not met, the participants were excluded from the study sample, as further detailed in Figure 1.

#### Training Regime

Sports training and its performance were taken into account but did not represent a determining factor due to our objective to analyze the normal evolution of athletes from different sports clubs. Thus, a training regime was not determined; rather, we took descriptive data regarding the weekly training. The data included information regarding the number of training sessions (at least three per week), the volume (at least 180 min per week), and the intensity reported as a percentage (%) of the maximum heart rate (HR_max_).

### 2.3. Applied Tests

All participants underwent the first testing stage, which included anthropometric and basal metabolic rate (BMR) measurements next to cardiac screening, which included both electro- and echocardiogram. During the second stage, the participants underwent the aerobic capacity assessment (VO_2peak_) during an incremental running test, carried out to the point of voluntary exhaustion.

#### 2.3.1. Anthropometric Analysis

We measured the body weight and the body height by using the ADE thaliometer (ADE Germany GmbH, Hamburg, Germany) under basal conditions during three successive repetitions, from which we extracted the average values. Further on, we used the HARPENDEN Professional Skinfold (Baty International Ltd., Burgess Hill, UK) to measure several skinfolds. The biceps, triceps, subscapular, suprailiac, and abdominal skin folds were measured and used next to age, weight, and height in Durnin and Womersley formula to determine body fat-free mass (% of the body weight and kg) and fat mass.

#### 2.3.2. Basal Metabolic Rate Measurement through Indirect Calorimetry Method

The basal metabolic rate (BMR) was measured early in the morning, on an empty stomach, before any other tests. We used the Cortex Metalyzer 3B equipment (Cortex Medical, Leipzig, Germany), which was calibrated with known O_2_ (15%) and CO_2_ (5%) concentrations and had predetermined RQ, VCO_2_, and VO_2_ variability ranges.

Every athlete was called to the measurement room, where they spent 15 min adapting to environmental conditions. The test lasted about 15 to 20 min and was automatically stopped when the steady-state period was reached. By following the measurement protocol, we focused on extracting information regarding energy expenditure (EE, kcal/day/hour/minute) in basal conditions, next to respiratory quotient (RQ), oxygen uptake (VO_2_, mL/min), carbon dioxide production (VCO_2_, mL/min), the use of carbohydrates (CHO, g/day) and fat (Fat, g/day), the respiratory frequency (Rf, c/min), minute ventilation (VE, L/min), and the tidal volume (VT, mL).

#### 2.3.3. Measuring VO_2peak_ during an Incremental Running Test

VO_2peak_ was measured during a second visit. We used the Cortex Metalyzer 3B, Pluto It Med treadmill (Nußdorf, Germany), and Polar H10 heart rate belt (Kempele, Finland), to perform an incremental exercise test, which was held until voluntary exhaustion.

Each participant showed up in the laboratory according to the testing schedule, and once present, she was once more informed of the means and methods of testing. The test was conducted on the Pluto It Med treadmill by following an adapted version of the Bruce testing protocol. The participants went through stages of 3 min in length, following a minimum speed of 2.74 km/h during the first stage and 18 km/h during the last stage, without any changes in the treadmill slope, due to a lack of everyday exercise specificity.

Before testing, the equipment was calibrated with known O_2_ (15%) and CO_2_ (5%) concentrations. The turbine was calibrated before each test with air volumes individually adapted to the age-related normal values. The test was ended if the RQ was equal to 1.10, if the oxygen uptake reached a plateau (±150 mL), or if the participant reached voluntary exhaustion. During the test, we measured oxygen uptake (VO_2_), oxygen uptake per heartbeat (VO_2_/HR), ventilatory equivalents for oxygen (VE/VO_2_), ventilatory equivalents for carbon dioxide (VE/VCO_2_), heart rate (HR), respiratory frequency (Rf), carbon dioxide production (VCO_2_), physiological dead space estimation (VD/VT(est), ventilation (VE), the ratio between maximal ventilation during exercise and maximum voluntary ventilation (VE/MVV), end-tidal partial pressure of oxygen (PetO_2_), end-tidal partial pressure of carbon dioxide (PetCO_2_), inspired and mixed expired PO_2_ (PEO_2_), inspired and mixed expired PCO_2_ (PECO_2_), metabolic equivalent (METs), fractional content of oxygen (FeO_2_), and fractional content of carbon dioxide (FeCO_2_).

By using the abovementioned data, we further applied the V-Slope method and determined both the anaerobic threshold (AT) and the respiratory compensation point (RCP).

### 2.4. Statistical Evaluation

The statistical evaluation was carried out with the GraphPad Prism 6.0 software (Graph Pad Software, San Diego, CA, USA). The level of significance was set at α = 0.05, while the following tests were used in the inferential statistics: the D’Agostino and Pearson omnibus normality test for data distribution, the Mann–Whitney test to prove the differences between two items, and the Spearman r test for assessing the relationship between two analyzed parameters. The data were presented by using the median value, the minimum/ maximum values, and the variation coefficient (CV).

## 3. Results

The median age in the study sample (*n* = 40 participants) was 15.85 years old. The median body height was 168 cm, the median body weight was 57.62 kg, and the fat mass was 18.34%, as seen in Table 1, which summarizes the demographic data of the study sample (*n* = 40).

*Sample 1* had a median training volume of 7.5 h during five out of seven days, unlike *Sample 2*, which had a median of 10 h during six out of seven days. The intensity reported was somewhat similar, with lower overall intensity for *Sample 1* (68% of HRmax) versus *Sample 2* (76% of HR_max_), taking into account the existence of two intensity workouts conducted at 85–90% of HR_max_ in *Sample 2.* Sample 1 had some intense daily activity through the specifics of the game but did not report submaximal activity of short or medium duration led by running, cycling, or any other form of training.

### 3.1. Comparative Analysis of the BMR Measurements in Both Sample 1 and Sample 2

The study sample had a theoretical BMR of 1446,54 kcal/day. Unlike the theoretical value, the measured BMR was 1734 kcal/day (+19.87%). In *Sample 1*, the theoretical BMR value was 1500 kcal (CV = 8.42%), and the measured BMR was 1767 kcal/day (2089 to 1569 kcal/day min to max). The BMR was attributed to an oxygen uptake of 0.24 L/min, 0.23 L/min VCO_2_, and 0.89 RQ.

The BMR for total body surface area (BMR/BSA) was 988 kcal, and the BMR per kg of lean body mass was 29.4 kcal (21.5–36 kcal min to max). In *Sample 2*, we calculated a theoretical BMR of 1392 kcal and measured a median BMR value of 1585 kcal/day (CV = 10.95%) (1403 to 2026 kcal/day min to max values). During the BMR measurement, the oxygen uptake was 0.22 L/min with 0.20 L/min VCO_2_ and 0.86 RQ, as illustrated in Table 2.

### 3.2. The Results of the Incremental Test in Both Sample 1 and Sample 2

In *Sample 1*, the AT point was reached at 1.57 L/min and 25 mL/min/kg VO_2_, where the VO_2_/HR was 9.5 mL/b, VE/VCO_2_ was 26.85 L/min, and VE/VO_2_ was 29.65 L/min. Minute ventilation was 50.45 L/min at 36 c/min BF and 1.32 L/min VT. In the same study sample, VCO_2_ was 1.51 L/min, RQ was 0.91, and the energy expenditure was 490.5 kcal/hour. As the intensity increased, oxygen uptake at RCP was 2.33 L/min and 35 mL/min/kg, while VO_2_/HR was 12 mL/b at 31.2 L/min VE/VO_2_ and 31.1 L/min VE/VO_2_.

In *Sample 2* the oxygen uptake at AT was 1.37 L/min and 25.5 mL/min/kg. Following the study group, VE/VO_2_ was 25.4 L/min, VE/VCO_2_ was 28.4 L/min, VE was 42 L/min, and VCO_2_ was 1.26 L/min. The oxygen uptake at the RCP point was 2.04 L/min and 35.5 mL/min/kg. The VO_2_/HR was 11.5 mL/b, while both VE/VO_2_ and VE/VCO_2_ reached 30.4 and 30.1 L/min at 68.55 L/min VE. A comparative analysis is carried out in Table 3, where AT and RCP points are compared between the two samples.

In *Sample 1*, VO_2peak_ was 2.38 L/min and 37.5 mL/min/kg, unlike *Sample 2*, which had 2.31 L/min (*p* = 0.402, Mann–Whitney U = 204) and 43 mL/min/kg oxygen uptake (*p* = 0.0001, Mann–Whitney U = 72). VE/VO_2_ was significantly different (*p* = 0.0002, Mann–Whitney U = 80) by reaching 31.3 and 36.1 L/min in *Sample 1* and *Sample 2*.

Similar results were obtained regarding VE/VCO_2_ (*p* = 0.0074, Mann–Whitney U = 126) due to 31.3 and 33.9 L/min in *Sample 1* and *Sample 2*, while RQ was as well different (1.05 vs. 1.09) in the two sample groups (*p* = 0.0001, Mann–Whitney U = 72). Differences were as well encountered regarding PetO_2_ in both samples (110.5 vs. 115.5 mmHg) (*p* = 0.0001, Mann–Whitney U = 62), next to PetCO_2_ (33.5 vs. 31.5 mmHg) following *p* = 0.0477, Mann–Whitney U = 156 statistical test results.

## 4. Discussion

We analyzed the differences in the AT, RCP, and VO_2peak_ values in the two samples, which included athletes from a team and an endurance sport. *Sample 1* included volleyball players, and *Sample 2* was made up of triathlon athletes. Against our expectations, the results showed a lack of difference regarding oxygen uptake at both AT and RCP, with some differences regarding respiratory efficiency at VO_2peak_. The results we obtained reflect a transition in public health, through the role of physical exercise and the expectations of each athlete in relationship to sports training or physical exercise. It appears that there are many cases where exercise does not produce the desired cellular changes due to the goals, means, and methods of exercise. This assumption seems to be found in everyday life, which is why underdeveloped countries have fewer and fewer high-performing athletes.

### 4.1. Age-Related Norms

Existing papers described the importance of daily physical exercise to improve cell metabolism, neuromuscular function, and cardiopulmonary capacity [27,28,29]; however, few research papers have investigated overtraining or physical underdevelopment induced by sports training in children [30,31,32], and under/overtraining cases are not low among children and should not be ignored. The topic becomes of interest as a result of the influence it has on the general population’s health, the number of children who participate in various forms of physical exercise, and the number of high-performance athletes.

By analyzing our data and considering that volleyball has an intense anaerobic exercise practice, the physiological and anthropometric differences between the two samples are not up to our expectations. There are still differences in both AT and RCP regardless of exercise specificity and physical exercise metabolism. For example, *Sample 1* reached 103% of VO_2norm_ (89 to 123% min to max), while *Sample 2* reached 116.5% of VO_2norm_ (98 to 129% min to max), which summarizes an aerobic capacity value that is above normal age values. Milenković et al. [33] published a paper on a research study conducted on handball players. In their paper, the participants had a higher oxygen uptake compared to volleyball players. However, both groups had a higher oxygen uptake than non-active individuals, but lower values compared to endurance athletes. Other papers [34] stated values between 33 and 48.1 mL/min/kg, which are somewhat similar to those in our study sample, which had an average oxygen uptake of 37 mL/min/kg in 17-year-old sportswomen. Unlike volleyball players, triathlon athletes had a higher aerobic capacity and therefore an improved AT. This result was confirmed early on in 10- and 16-year-old sportswomen who reached 1.5 to 2.2. L/min oxygen uptake, similar to young female runners aged between 10 and 16 years old who had up to 54 mL/min/kg oxygen uptake. However, our study samples failed to exceed 2.3 L/min and 43 mL/min/kg VO_2_.

#### Anthropometric Results

According to Lidor et al.’s [34] review, the female volleyball player is between 164.3 and 187 cm tall, with a 62.5 and 75.1 kg body weight and 23.4% mean body fat. Similar results were published by Güzel et al. [35], who reported that fat mass in young adolescent sportswomen is between 26 and 31%, unlike our sample value, which had 18 and 20%. However, non-active adolescent females tend to have even higher values than those stated in our paper, and this, for the most part, sums up the difference between physically active and inactive individuals. However, the development of the fat-free mass is deficient, probably also as a result of the daily food intake.

### 4.2. Incremental Exercise Test Results—A Comparison with the Literature Report

Oxygen uptake represents a stable scientifically accepted method to measure oxygen uptake, which is considered a high-performance parameter and a selection criterion in various sports. Hottenrott et al. [36] proved that physical exercise can lead to significant changes in aerobic capacity, whereas movement proficiency is strongly improved by organized physical activity.

Following Birat et al.’s study [37], which was conducted on a sample of 12 prepubertal, 12 adult, and 13 male endurance participants, we observed that children can recover in a shorter period not because of muscular endurance but due to energy metabolism. However, sports practice and individual physiological characteristics can significantly influence these measures, which were proven in small groups. Further, Shete et al. [38] followed a group of female athletes and non-athletes aged between 17 and 22 years and reported mean differences of 16 mL/min/kg between groups, against 5.5 mL/min/kg measured in our study sample. Other authors [39] reported on female volleyball athletes who were aged 17.1 years old, with values between 41.09 and 42.56 mL/min/kg, going against our outcomes, which failed to exceed 37.5 and 43 mL/min/kg. However, most results will differ depending on sports practice, age, and gender.

As a result of the variability between our study groups and the results of other research, we included other parameters regarding respiratory efficiency. Salazar-Martínez et al. [40] studied adult male ventilatory efficiency and concluded that VE/VCO_2_ may represent a fixed variable that has no significant change between athletes. However, our study conducted on young female athletes had differences in both VE/VCO_2_ and VE/VO_2_. More specifically, lower ventilatory efficiency was seen next to lower VO_2peak_ values in Sample 2, unlike in Sample 1. The differences were preserved over PetO_2_ and PetCO_2_ during a physical exercise held at VO_2peak_ intensity. According to Salazar-Martínez et al. [40], higher PetCO_2_ can induce a higher tidal volume and lower respiratory frequency, which is considered a highly efficient physiological adaptation. This result seems to be applicable, considering that an increased mean value was observed in *Sample 1*, which had a comparative increase in PetO_2_, a comparative decrease in VCO_2_, and a reduction of EE at RCP. However, none of the reported measures were significantly statistically different at RCP (*p* > 0.05), but the average values were slightly different.

Differences were measured at VO_2max_ exercise intensity, where statistical differences were obtained regarding PetO_2_, VE/VO_2_, VE/VCO_2_, METS, and VO_2max_ relative value. However, Datta et al. [41] proved in male participants that VE/VCO_2_ and FeO_2_ have less importance on the physiological variables measured during exercise, due to changes in RER, O_2_ uptake, and CO_2_ production. Furthermore, the differences regarding the metabolic equivalent are less important by taking into account oxygen uptake, similar to Cristi-Montero’s [42] statement, which concluded that heart rate, unlike METS, can represent a more important indicator of physical exercise intensity. Thus, in the study groups, the aerobic capacity of athletes between the ages of 14 and 16 increased, but the lack of induced respiratory efficiency is still observed due to higher metabolic costs. However, we wonder if this is progress over a sedentary sample group, namely a slight decrease in inactive mass and a sensible increase in aerobic capacity (5–15%), with a lack of ventilatory efficiency, attributed according to age, at high intensity. Moreover, if these are the most important benefits that physical exercise can bring to a group of teenagers, we need to rethink the methodology and means of sports training to form high-performance athletes or/and healthy adults with social contribution and healthy habits.

We must consider the study limitations, among which the number of participants may bring different results depending on the study sample, the exercise capacity, and individual performance. Future research should include a detailed analysis of the training by including data regarding the volume, intensity, and physical exercise specificity. The study of cardiac mechanical activity would be important to see the adaptation induced by submaximal exercise, while strength development should be evaluated to determine the anaerobic potential. Finally, the inclusion of male participants may add to the published results.

## 5. Conclusions

By comparing a team sport and triathlon, we might expect different results regarding exercise capacity. However, physical training cannot create special physiological abilities, but it can influence the existing ones. In some cases, training can positively influence; in other cases, due to a low exercise capacity and a lack of adaptation, the improvement of motor qualities will be nonexistent, causing rather a functional overload and lack of development.

The athletes included in the study sample had no differences regarding oxygen consumption at AT and RCP. However, differences were seen in oxygen consumption at VO_2peak_. The contribution of physical activity is observed in both groups, taking into account the oxygen uptake, as compared to the literature reports of non-active individuals. Sample 1 had a lack of efficiency and increased metabolic costs at VO_2peak_ exercise intensity, as compared to Sample 2. These results and the underestimation of parameters may be due to sports training. Further research will have to include more physiological variables to observe the physiological adaptation to physical exercise and its specificity.

## Figures and Tables

**Table 1:** Descriptive analysis regarding the study group.

Parameter	Sample 1	CV, %	Sample 2	CV, %	*p*	Mann–Whitney U
Age	16	10,21	15.5	12,5	0.5199	50
Body weight, kg	61,5	19,75	53,55	16,1	0.0007	96
Body height, cm	177	4,47	160	4,68	0.0001	30
Fat mass, %	20	16.33	18.55	34.79	0.5334	140

Legend: kg—kilograms; cm—centimeters; %—percentage; *p* = probability level.

**Table 2:** A comparative analysis regarding the BMR measurement in Sample 1 and Sample 2.

Parameter	Sample 1 Median (Min to Max)	Sample 2 Median (Min to Max)	*p*	Mann–Whitney U
VO_2,_ mL/min	0.245 (0.23 to 0.29)	0.22 (0.20 to 0.29)	0.0087	115,5
VCO_2,_ mL/min	0.23 (0.2 to 0.27)	0.20 (0.17 to 0.26)	0.0008	66
RQ	0.89 (0.83 to 0.94)	0.86 (0.81 to 0.89)	0.0058	86
Fat, g/day	62 (28 to 92)	72.5 (52 to 113)	0.183	134
CHO, g/day	277 (173 to 362)	199.5 (150 to 318)	0.0005	60
BMR	1767 (1569 to 2089)	1585 (1403 to 2026)	0.0074	88
Theoretical BMR, kcal/day	1500 (1392 to 1800)	1392 (1248 to 1488)	0.0001	72
Difference between BMR and _t_BMR, %	84.35 (70.09 to 104.62)	84 (73.45 to 94.05)	0.197	126
BF, rc/min	14 (11 to 18)	18 (11 to 20)	0.0191	100
VT, L	0.547 (0.45 to 0.79)	0.45 (0.31 to 0.65)	0.0087	90
VE, L/min	8.2 (6.9 to 9.9)	7 (6.4 to 8.7)	0.0051	84
BMR/BSA	988 (910 to 1230)	1084 (987 to 1225)	0.0201	100
BMR/kg_Lean	29.4 (21.5 to 36)	32.85 (28 to 43)	0.0003	56

Legend: VO_2_—oxygen uptake, VCO_2_—carbon dioxide production, RQ—respiratory quotient, CHO—carbohydrates, BMR—basal metabolic rate, BF—breath frequency, VT—tidal volume, VE—ventilation, BSA—body surface area, tBMR – theoretical basal metabolic rate, *p*—probability level.

**Table 3:** A Comparative analysis regarding AT and RCP data in Sample 1 and Sample 2.

	Parameter	Sample 1 Median (Min to Max)	Sample 2 Median (Min to Max)	*p*	Mann–Whitney U
AT	VO_2,_ L/min	1.57 (0.43 to 2.5)	1.37 (1.04 to 1.91)	0.190	184
	VO_2,_ ml/min/kg	25 (17 to 30)	25.5 (23 to 42)	0.172	182
	VO_2_/HR, ml/b	9.5 (7 to 14)	9 (6 to 12)	0.573	216
	VE/VO_2_, L/min	26.85 (21.9 to 32.7)	25.4 (20.5 to 30.9)	0.146	178
	VE/VCO_2_, L/min	29.65 (24.2 to 34.9)	28.4 (23.8 to 33.6)	0.146	178
	VCO_2_, L/min	1.51 (0.8 to 2.24)	1.26 (0.87 to 1.65)	0.075	164
	RQ	0.91 (0.87 to 0.94)	0.91 (0.82 to 0.93)	0.644	220
	PetO_2_, mmHg	105 (98 to 113)	106 (101 to 113)	0.990	240
	PetCO_2,_ mmHg	34.5 (29 to 42)	37 (31 to 40)	0.081	166
	EE, kcal/hour	490.5 (127 to 749)	402.5 (295 to 566)	0.111	172
RCP	VO_2,_ L/min	2.33 (1.58 to 3.06)	2.04 (1.52 to 2.41)	0.173	162
	VO_2,_ ml/min/kg	35 (26 to 40)	35.5 (31 to 55)	0.076	146
	VO_2_/HR, ml/b	12 (8 to 16)	11.5 (8 to 13)	0.284	174
	VE/VO_2_, L/min	31.2 (23.5 to 34.7)	30.4 (25.6 to 37.4)	0.423	184
	VE/VCO_2_, L/min	31.1 (23.4 to 34.8)	30.1 (25.5 to 38.7)	0.143	158
	VCO_2_, L/min	2.34 (1.51 to 3.07)	2.05 (1.52 to 2.42)	0.189	164
	RQ	1 (0.96 to 1)	1.00 (0.97 to 1.03)	0.049	144
	PetO_2_, mmHg	111 (99 to 114)	112 (106 to 115)	0.581	194
	PetCO_2,_ mmHg	32 (30 to 46)	35 (29 to 37)	0.115	154
	EE, kcal/hour	697 (470 to 915)	609.5 (454 to 717)	0.158	160

Legend: Min—minimum; Max—maximum; CV—Coefficient of variation; %—percentage; g/day—grams per day.
